# Medial to posterior release procedure after failure of functional treatment in clubfoot: a prospective study

**DOI:** 10.1007/s11832-016-0728-6

**Published:** 2016-03-31

**Authors:** Nicolas Bocahut, Anne-Laure Simon, Keyvan Mazda, Brice Ilharreborde, Philippe Souchet

**Affiliations:** Pediatric Orthopedic Department, Robert Debré Hospital, Assistance Publique-Hôpitaux de Paris (AP-HP), Paris Diderot University, 48 Bd Sérurier, 75019 Paris, France; Pediatric Orthopedic Department, Marcel Sembat Clinic, Générale de Santé, 105 Avenue Victor Hugo, 92100 Boulogne Billancourt, France

**Keywords:** Clubfoot, Functional method, Residual, Recurrent, Selective soft-tissue release

## Abstract

**Purpose:**

Two conservative techniques for clubfoot treatment are still being debated and depend upon the institution’s expertise. For >40 years, the current institution has been a pioneer in the development of the physiotherapy method; however, some severe deformities remain resistant to this method which causes pain, sprains, and difficulties wearing shoes. Therefore, a surgical approach was developed simultaneously for the treatment of these residual or recurring clubfeet. The procedure reproduces the same chronological steps by performing forefoot derotation before correcting hindfoot equinus. The aim of the current study was to assess the results of this surgical technique.

**Methods:**

All clubfeet undergoing surgery between October 1995 and February 2009 were prospectively included. Initial severity was based on Dimeglio’s classification and final outcomes on the International Clubfoot Study Group (ICFSG) outcome evaluation system. Last follow-up results were assessed by physical examination and radiographs.

**Results:**

A total of 137 patients with severe clubfeet (mean Dimeglio score 12.0) underwent surgery. At the mean follow-up of 10.8 years, mean ICFSG score was 4.3 (range 0–23), and 12 % required revision surgery. The rate of undercorrection and overcorrection was low (17 pes-plano-valgus ft and 11 ft with undercorrection). Eight feet had a fixed deformity.

**Conclusions:**

Severe deformities are more resistant to conservative techniques even for institutions with large experience. These deformities require further treatment, including surgery if necessary. The medial to posterior soft-tissue release is a valuable technique with stable results.

**Level of evidence:**

Level IV.

## Introduction

Over the past 20 years, conservative treatment has been the first option for the management of clubfeet. Two techniques are largely debated—the Ponseti method (PM) and the French functional method (FFM) with equal results [1–3]. Literature reported range from 3 to 55 % in recent and older publications for clubfeet treated by a conservative technique [3–9]. The current institution has been a pioneer in the development of the FFM and has trained physiotherapists for >40 years [2, 10]. Despite this expertise, some severe deformities remain resistant and require further treatment [11, 12]. As reported, a non-plantigrade and misaligned foot causes pain, sprains, and difficulties wearing shoes and results in long-term degenerative lower limb osteoarthritis in adulthood [12, 13].

The medial to posterior soft-tissue release technique has been simultaneously developed at our institution for the treatment of resistant clubfeet, defined by the persistence or the recurrence of the following deformities—forefoot adduction and supination, hindfoot medial rotation and equinus [14, 15]. The procedure is based on the same chronological steps as PM and FFM, with the aim being to derotate the forefoot before reduction of hindfoot equinus [1, 2, 16].

This surgical approach differs from Turco’s and Carroll’s procedures [17, 18]. Prior techniques are based on extensive soft-tissue release including lengthening of plantar flexor muscles. Numerous studies had demonstrated a lack of good long-term results for these techniques, with high rates of revision surgery (21–87 % at 2–10 years postoperatively) and poor functional outcomes according to variable scoring systems [8, 19–25]. However, few authors have reported results using more selective approaches [26–28].

The aim of the current study was to assess the results of the medioposterior soft-tissue release technique in a large cohort of clubfeet followed prospectively from birth. The hypothesis was that this surgical procedure provides an anatomical correction with few fixed residual deformities and good functional results.

## Materials and methods

### Patients

Data were collected after the parents of the children signed an informed consent approved by the Institutional Review Board. Children receiving care in the pediatric orthopedic department for a clubfoot deformity were prospectively enrolled from birth between 1995 and 2009. Initial clubfoot severity was assessed by Dimeglio’s classification [29]. All the patients were immediately treated by the FFM. Similar to the PM, the aim is to obtain forefoot derotation in order to reduce medial talonavicular joint dislocation before correction of hindfoot equinus. After treatment initiation, patients were seen by the same experimented senior surgeon at the age of 6 weeks, and at 3, 9, 12, 18 and 24 months and then once per year. For each visit, dorsoplantar and lateral foot radiographs (simulated weight-bearing X-rays before walking age and standing X-rays after walking age) were performed. A combination of the four following criteria was the only indication for surgery—(1) a non-plantigrade foot posture on visual observation of gait, (2) dorsiflexion <10°, (3) a lack of talocalcaneal angle on dorsoplantar radiographs, and (4) a misalignment of the talo-first metatarsal angle or of the calcaneo-fifth metatarsal angle on the dorsoplantar view radiographs. All operated feet had these four components that were either uncorrectable after manipulation or recurred during follow-up. Since the navicular bone is not ossified before 4 years of age, misalignement of the talo-first metatarsal angle indicates navicular bone medial subluxation. The FFM was never interrupted until surgery and no patient received additional conservative treatment by the PM, since surgeons were not trained for this technique. All patients with a suspicion of secondary etiology were referred to a pediatric neurologist and geneticist (Table 1). Idiopathic clubfeet (ICF) and non-idiopathic clubfeet (NIFC) were analyzed in the current study to avoid removing the worst results and to stay as unbiased as possible.

**Table 1:** Non-iodiopathic clubfeet etiologies

Etiology	No. of feet	No. of patients	No. of bilateral surgery
Foot dorsiflexion muscles paralysis^a^	10	7	3
Arthrogryposis	12	7	5
Behavioral disorders—autism	6	4	2
Musculoskeletal anomalies	5	3	1
Polymalformative syndrome	3	2	1
Amniotic band syndrome	4	3	1
Total	40	26	13

^a^Patients with an isolated paralysis of foot dorsiflexion muscles for whom a precise etiology was not diagnosed

### Surgical procedure

The medioposterior soft-tissue release technique is performed through a unique medial approach and follows different steps according to the pathophysiology of the deformity [15, 16]. The procedure always consists first of an anteromedial release followed by a posterolateral release if necessary, using a scapel blade. Indeed, the surgeon avoids any detachment of non-involved tissues in order to prevent extensive postoperative fibrosis. The anteromedial release (adductor hallucis excision, plantar fascia release, tibialis posterior lengthening and talonavicular capsulotomy) reduces the talonavicular joint dislocation and medial rotation of the calcaneoforefoot unit [30]. The deformity is reduced when the navicular bone is in front of the talar head. The navicular bone is no longer rotated internally and the first metatarsal is perfectly aligned with the talus. The posterolateral release corrects the equinus. If a residual equinus persists despite Achilles tendon *Z*-lengthening, a posterior ankle arthrotomy is performed through the same approach. Scissors are then introduced and pushed as far as the lateral malleolus and fibularis tendon sheath, to dissect the lateroposterior node. At that stage, 10° of ankle dorsiflexion is obtained. Posterior subtalar joint arthrotomy is not indicated for the correction of hindfoot equinus since only the tibiotalar joint has sagittal plane movement. Moreover, the talocalcaneal ligament is particularly respected to avoid overcorrection with lateral translation and valgus induced by destabilization of the subtalar joint [31]. The tibialis posterior and Achilles tendon are sutured at the end of the procedure, with the foot in a neutral position. To maintain the correction, one *k*-wire is introduced under fluoroscopic control from the first metatarsal bone to the talus and across the talonavicular joint. The *k*-wire maintains the alignment of the foot. Patients are immobilized for 6 weeks in a non-bearing long-leg cast with the knee flexed at 90° in order to maintain ankle and foot dorsiflexion. The pin is removed 6 weeks after surgery.

### Outcomes evaluation

Postoperatively, the same surgeon saw children at 6 weeks, and at 3 and 12 months, and then once per year until skeletal maturity. At each visit, dorsoplantar and lateral radiographs were performed. The physical examination consisted of visual observation of gait to assess morphological and dynamic alignment of the foot and of measurements of ankle, subtalar and forefoot range of motion (ROM). Final outcomes were evaluated by the ICFSG score with a minimum postoperative follow-up of 5 years (Table 2). Results are classified as very good (0–5), good (6–15), fair (16–30) and poor ≥30 [10, 32, 33]. The procedure is performed after the age of 6 years, since mature gait is not fully developed before that age [32]. Two junior surgeons reviewed clinical and radiological data in order to detect inconsistencies in recording. Recording by an independent observer was not performed.

**Table 2:** International Clubfoot Study Group (ICFSG) outcome evaluation system

Morphology	Score		
Hindfoot			
Varus or valgus	0	1 (10°)	2 (>10°)
Equinus or calcaneus	0	1 (10°)	2 (>10°)
Midfoot			
Supination or pronation	0	1 (10°)	2 (>10°)
Adduction or abduction	0	1 (10°)	2 (>10°)
Global alignment of the foot			
Medial or lateral rotation (thigh–knee–foot angle)	0	1 (10°)	2 (>10°)
Pes cavus or flatfoot	0	1 (10°)	2 (>10°)
Maximum			/12

Functional evaluation	Score		
Passive motion			
Ankle			
Dorsiflexion	0	1 (0°)	2 (negative)
Plantarflexion	0	1 (10°)	2 (<10°)
Subtalar varus-valgus	Flexible/stiff	0	1
Midtarsal pronation–supination	Flexible/stiff	0	1

Muscle function	Score		
	Normal	Moderate	Severe
Jones’ classification	(4, 5)	(3)	(0, 1, 2)
Triceps surae			
Flexor digitorum			
Flexor hallucis longus			
Tibialis posterior			
Extensor digitorum			
Extensor hallucis longus			
Tibialis anterior			
Fibularis longus and brevis			

Dynamic function	None	Positive	
Gait			
Intoeing (medial rotation)	0	1 (10°)	2 (>10°)
Talus	0	1 (10°)	2 (>10°)
Equinus	0	1 (10°)	2 (>10°)
Dynamic supination	0	1 (10°)	2 (>10°)
Limping	0	1	
Ability to run	1	0	
Ability to jump	1	0	
Shoe wear		0 normal	1 abnormal
Heel walking or toe walking		0 yes	1 no
Pain			
No pain	0		
Pain during activities	1		
Pain during sports	2		
Permanent pain	3		
Maximum			/36

Radiological evaluation	Score	
	Normal	Abnormal
Standing anteroposterior views		
Talocalcaneal angle	0	1
Calcaneocuboid alignment	0	1
Calcaneo-fifth metatarsal axis	0	1
Talo-first metatarsal axis	0	1
Talonavicular position	0	1
Standing lateral views		
Talocalcaneal angle	0	1
Tibiocalcaneal angle	0	1
Talonavicular position	0	1
Talo-first metatarsal axis	0	1
Calcaneo-fifth metatarsal axis	0	1
Flat top talus	0	1
Standing ankle anteroposterior views (alignment of lateral and medial malleoli shank external rotation)	0	1
Maximum		/12
Total		60

### Statistical analysis

Statistical analyses were performed using JMP 10.0 (SAS Institute Inc., Cary, NC, USA). Numeric data were expressed as mean ± standard error of the mean (SEM). A Shapiro–Wilk test was performed to assess data distribution. Group comparisons used a two-tailed Student *t* test for variables with normal distribution. A 2-sample Wilcoxon test was performed for non-parametric data. Fisher’s exact test was used for qualitative data. Statistically significant results were accepted as valid with a significance of *p* < 0.05.

## Results

### Patients

**Fig. 1:**
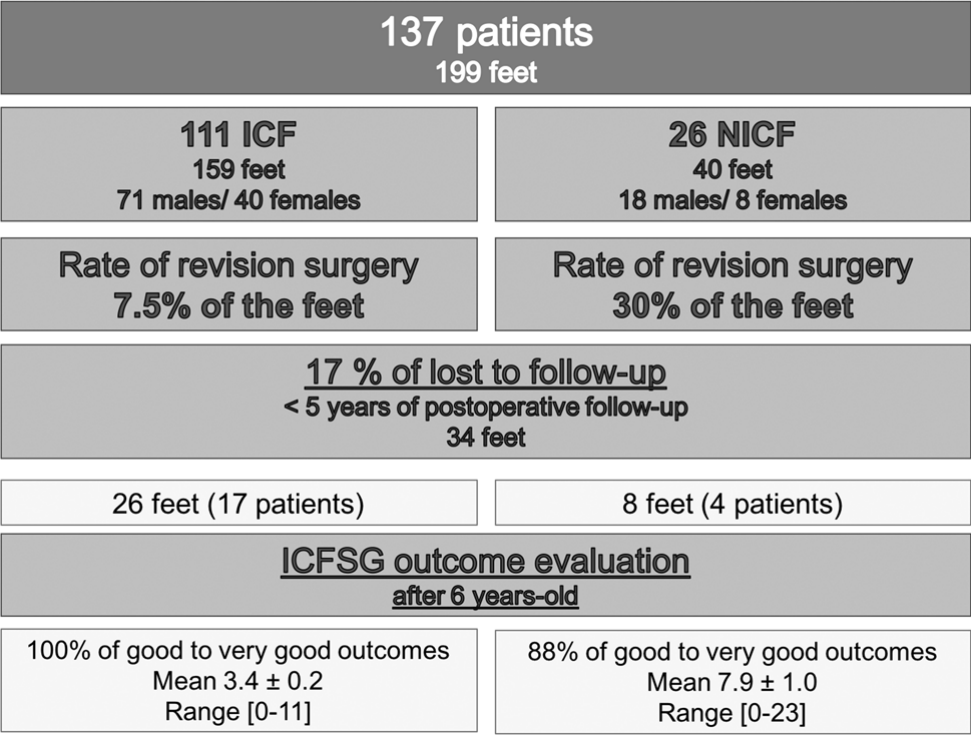
Flow-chart of the study group

Between 1995 and 2009, 359 patients (513 ft) were treated by the FFM. A total of 137 children (199 ft) underwent surgery (80 % ICF vs 20 % NICF) (Fig. 1). These patients had a significantly (*p* < 0.0001) higher Dimeglio’s score at birth and a higher ICFSG score compared to patients successfully treated by the FFM. Initial assessment was performed at an average age of 22.9 ± 3.6 days. Mean Dimeglio score was 12.0 ± 0.2 (grade I, 1 ft; grade II, 50 ft; grade III, 123 ft; and grade IV, 25 ft).

### Surgical procedure

The mean age at surgery was 1.4 ± 0.05 years (range 4.3 months to 4.7 years) with two peaks—one at 12–15 months (52 cases) and one at 18–24 months (41 cases).

### Global cohort

The average follow-up was 10.8 ± 0.2 years (range 5.1–18.5 years) with a mean age at follow-up of 12.3 ± 0.3 years (range 6–19.5 years). Twenty-one patients (34 ft) were lost to follow-up. Mean ICFSG score was 4.3 ± 0.3 (range 0–23). The scores were very good, good and fair in 70 % (ICF 103 ft vs NICF 12 ft), 27.7 % (ICF 30 ft vs NICF 18 ft), and 2.3 % (NICF 4 ft) of cases, respectively. There were no poor results.

Mean passive tibiotalar ROM was 30 ± 1.2° (range 0°–60°). Subtalar joint and forefoot pronosupination were stiff in 45 % (73 ft) and 29.7 % (48 ft) of cases, respectively. All the patients had a normal assessment on visual observation of gait and no limping; a painless walk was found in 97.5 % of cases (5 patients had pain).

**Fig. 2:**
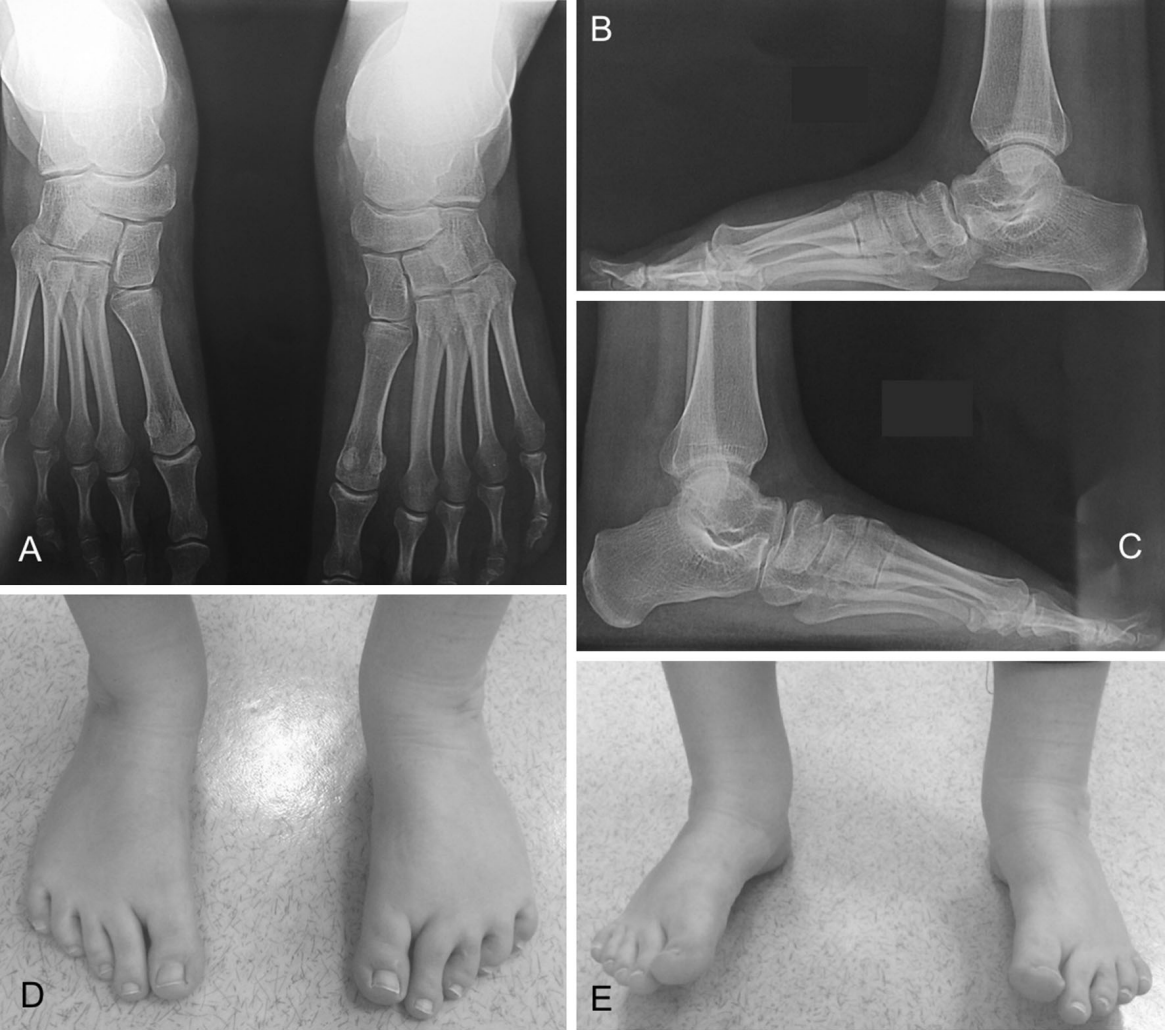
Idiopathic bilateral clubfoot at 18.5 year follow-up. Very good outcome (ICFSG score: 2). Calcaneoforefoot unit is derotated (**a**), talar dome is harmonious (**b**, **c**). Persistence of dynamic supination while walking on heels (**e**)

Fifty-nine feet had no morphological anomalies (Table 3). For 157 feet (95 %), deformities were dynamic, while walking on heels or toes and were isolated in 41.8 % of cases (69 ft) (Fig. 2).

**Table 3:** Morphological misalignment at follow-up

Variables	No. of feet (*n* = 165)
Overcorrection (total: 32 ft) 19.4 %	
Planus	30
Valgus	26
Supinatus	41
Undercorrection (total: 47 ft) 28.5 %	
Varus	7
Equinus	2
Adductus	15
Cavus	36
Internal rotation	3

Eight feet (5 %) had a fixed deformity

Percentages of over- and undercorrection correspond to either isolated or combined anomalies. Among the cohort, 17 feet were with planus and valgus, 4 feet relapsed and 11 were with cavus and adduction

Radiographic measurements were considered normal for all measurements in 57 % of the feet (94 ft) (Table 4). Forty-three feet (26 %) encountered one radiological anomaly. The most common anomaly was a misalignment of the talo-first metatarsal angle on lateral side views, associated with planus (9 ft), cavus (20 ft), forefoot supination (5 ft), hindfoot valgus (1 ft) or valgus (1 ft).

**Table 4:** Weight-bearing foot radiographic anomalies

Variables	Abnormal (*n*)
Standing anteroposterior views	
Talocalcaneal angle	12
Calcaneocuboid alignment	0
Calcaneo-fifth metatarsal axis	2
Talo-first metatarsal axis	2
Talonavicular position	2
Standing lateral views	
Talocalcaneal angle	4
Tibiocalcaneal angle	4
Talonavicular position	15
Talo-first metatarsal axis	45
Calcaneo-fifth metatarsal axis	0
Flat top talus	20
Standing ankle anteroposterior views (alignment of lateral and medial malleoli shank external rotation)	5

No precise angle measurements were performed since 10° precision is not reliable. Angles were considered as normal or abnormal according to ICFSG score

*n* number of feet

### Outcomes of NICF

**Fig. 3:**
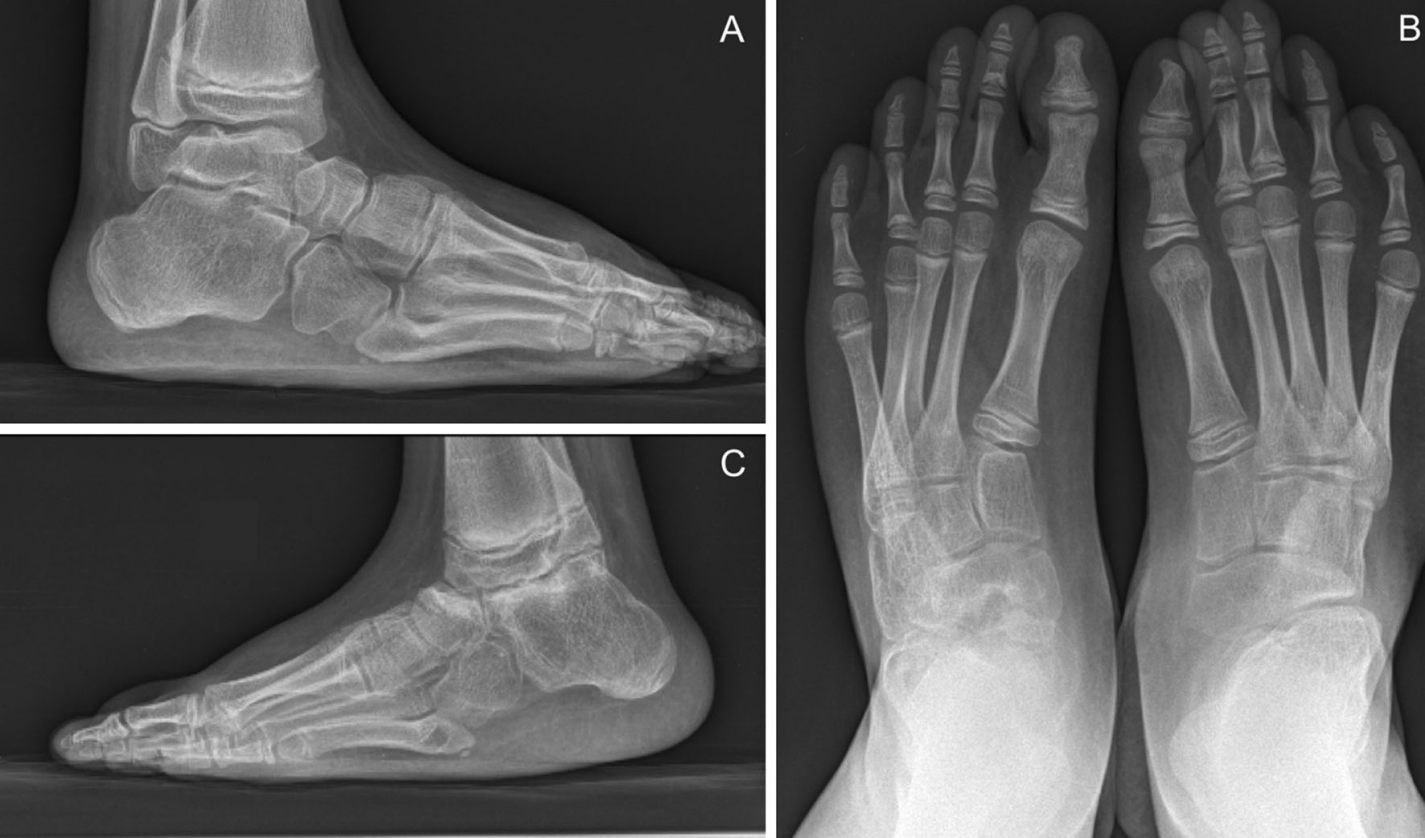
Non-idiopathic bilateral clubfoot secondary to arthrogryposis at 12-year follow-up. Fair result (worst ICFSG of the cohort: 23). A talectomy was performed 7 years after the first surgery on the* left side* (**b**). Fixed deformity with persistence of forefoot adduction, a lack of lateral derotation of the calcaneoforefoot unit (**c**) and forefoot supination (**a**)

As expected, the worst results were found with NICF (ICFSG: ICF 3.4 ± 0.2 vs NICF 7.9 ± 1.0, *p* = 0.0001). The prevalence of severe deformities at initial assessment was significantly higher for NICF (*p* = 0.04) with 8 feet at grade IV (20 %) versus 17 for ICF (10.7 %). Fair results were only found for NICF (4 ft); these were secondary to arthrogryposis and required revision surgeries (Fig. 3). Passive ROM was significantly lower in NICF (*p* < 0.00001) with a mean ankle ROM of 15.8 ± 2.4° versus 33.4 ± 1.2° for ICF and with subtalar and forefoot stiffness in 77 % (24 ft) and 65 % (20 ft) of cases, respectively.

### Descriptive analysis of the worst results

Twelve percent of the feet (24 ft) required revision surgery for relapsing (mean delay after first surgery 6 ± 0.7 years; range 2.4–13.6 years). Various procedures were performed (12× calcaneal derotation osteotomies, 5× Cahuzac procedures, 3× medioposterior release, 2× lapidus procedures, 1× triple arthrodesis and 1× talectomy). The calcaneal derotation osteotomy consisted of a curvilinear and extra-articular osteotomy for the correction of persistent medial rotation in older children with a stiffened subtalar joint. The Cahuzac procedure is a percutaneous technique which consists of a medial cuneiform osteotomy associated with the osteotomies of the second, third, and fourth metatarsals and cuboid bone for the correction of forefoot adduction. A second revision surgery was performed for three patients (1 NICF, 2 ICF). One patient required a third revision (triple arthrodesis). This patient who was affected by severe bilateral arthrogryposis required two revision surgeries on the left side and three on the right side.

Eight feet (5 %) had a fixed deformity (7 forefoot supination (5 ICF and 2 NICF) and 1 NICF with a cavus). Seventeen feet (10.3 %; 2 NICF and 15 ICF) had an overcorrection (pes-planus and hindfoot valgus), of which 12 had in addition a forefoot supination. Eleven feet were undercorrected—4 feet relapsed (2 NICF and 2 ICF) and 7 feet had a dynamic cavus and forefoot adduction.

There was no case of postoperative bone infection. Rate of skin necrosis was low (seven cases) and treated by controlled wound healing.

## Discussion

Treatment of clubfoot has been largely modify over the past couple of decades by the introduction of the PM and the FFM [1, 2]. However, some deformities remain resistant to conservative treatment, even for trained and specialized teams. Bad outcomes depend on multifactorial parameters [3, 10, 34–36].

In the current study, >70 % of patients had a severe deformity (grade III and IV) at birth and were therefore more resistant to well-conducted non-surgical treatment. Goldstein et al. recently demonstrated that a high Dimeglio score at birth is a predictive variable for surgery [11].

Management of relapsing deformities remains controversial. The physician has to decide whether to continue with non-surgical treatment, including combining techniques or to perform surgery.

Dunkley et al. recently showed low efficiency of repeat casting, with 86 % of patients relapsing after failure of the PM [37]. A study by McKay et al. found repeat casting and bracing for late relapsing failed in 94 % of cases [34]. As suggested by Richards et al., some patients do not respond well to conservative approaches despite additional attempts [36]. Therefore, surgery should not be avoided if necessary.

However, the type of surgical approach is not well defined. The medial to posterior release technique has been developed for the treatment of resistant clubfeet [15]. The technique is consistent with manipulations performed during both conservative methods by performing forefoot derotation before correction of hindfoot equinus [1, 2]. This approach should not be confused with Turco’s and Carroll’s techniques, which are extensive procedures associated with a global lengthening of all plantar flexor muscles with extensive arthrotomy [16, 17]. Burger et al. recently showed the necessity of conserving the talocalcaneal ligament to avoid overcorrection [31].

In the current study, global results based on the ICFSG score were better than previously reported [19–21, 23, 26–28, 38]. Regarding clinical outcomes, hind- and midfoot ROMs were limited. These results are consistent with previous reports (range 15°–27°) [26, 28]. As demonstrated by Wallander et al., joint foot ROM does not influence long-term function and osteoarthritis [39]. In their 60-year follow-up study, clubfeet treated either by extensive surgical procedures or by conservative treatment had a low rate of ankle and talonavicular severe osteoarthritis (8 and 12.4 %, respectively) with 50 % of very good and good functional results. Clubfeet will never be strictly normal, whatever the treatment. Some feet in the present study had a residual dynamic supination, which is a classic indication for tibialis anterior transfer; this procedure was not performed at the time of the study and is now discussed.

The rate of surgery (39 %) was high after the FFM in the present study. As demonstrated by Chotel et al., the FFM may be less efficient than the PM [40]. Conversely, Richards et al. showed that both techniques have a similar rate of residual deformities (94.4 and 95 % of initial success after PM and FFM, respectively), with a similar rate of recurrence (37 and 29 % early relapsing for PM and FFM, respectively) with 22 and 27 % of surgical procedures, even after combining conservative techniques [36]. Changes are currently performed in order to decrease the need for surgical procedures (Achilles tendon lengthening, long-leg braces) after FFM.

## Limitations

The first limitation of our study was the shorter follow-up time compared to previous reports and longer follow-up should be performed to detect late relapsing [5, 19–21, 23, 28]. However, compared to extensive posteromedial release, revision surgery was already performed 10 years after surgery [19, 20, 39]. Furthermore, the present study shows a low rate of revision surgery compared to the literature, where rates ranged between 21 and 87 % [8, 19, 25, 38].

The second limitation was that the same senior surgeon performed both initial and final assessment. Although a final assessment by an independent examiner would have limited the risk of bias, few patients were lost to follow-up and the assessment was consistent over the study period. Moreover, two junior surgeons searched the data in order to detect inconsistencies, which were reviewed.

The development of non-surgical techniques has decreased the need for clubfoot surgery. Nevertheless, severe deformities are more resistant to conservative techniques even for institutions with large expertise. The medioposterior release is performed according to the pathophysiology of the clubfoot deformity and is a valuable technique with stable results, regardless of the underlying pathology. Extended follow-up of the cohort is currently ongoing to further assess the outcomes at skeletal maturity and in early adulthood.
